# Determination of the packing fraction in photonic glass using synchrotron radiation nanotomography

**DOI:** 10.1107/S1600577516012960

**Published:** 2016-10-06

**Authors:** Malte Ogurreck, Jefferson J. do Rosario, Elisabeth W. Leib, Daniel Laipple, Imke Greving, Felix Marschall, Arndt Last, Gerold A. Schneider, Tobias Vossmeyer, Horst Weller, Felix Beckmann, Martin Müller

**Affiliations:** a Institute of Materials Research, Helmholtz-Zentrum Geesthacht, Max-Planck-Strasse 1, 21502 Geesthacht, Germany; b Institute of Advanced Ceramics, Technical University Hamburg-Harburg, Denickestrasse 15, 21073 Hamburg, Germany; c Institute of Physical Chemistry, University of Hamburg, Grindelallee 117, 20146 Hamburg, Germany; d Institute of Microstructure Technology, Karlsruhe Institute of Technology, Hermann-von-Helmholtz-Platz 1, 76344 Eggenstein-Leopoldshafen, Germany

**Keywords:** nanotomography, photonic glass, X-ray microscopy

## Abstract

Photonic glass is a material class that can be used as photonic broadband reflectors, for example in the infrared regime as thermal barrier coating films. Photonic properties such as the reflectivity depend on the ordering and material packing fraction over the complete film thickness of up to 100 µm. Nanotomography allows acquiring these key parameters throughout the sample volume at the required resolution in a non-destructive way. By performing a nanotomography measurement at the PETRA III beamline P05 on a photonic glass film, the packing fraction throughout the complete sample thickness was analyzed. The results showed a packing fraction significantly smaller than the expected random close packing giving important information for improving the fabrication and processing methods of photonic glass material in the future.

## Abstract 2

Synchrotron radiation nanotomography has been used to quantify the packing fraction in a photonic glass sample.

## 1. Introduction   

Photonic glasses are a relatively novel photonic material class (García *et al.*, 2007 ▸) which have a number of applications, for example as random lasing materials (Gottardo *et al.*, 2008 ▸; García *et al.*, 2010 ▸) or as thermal barrier coatings (TBCs) (Dyachenko *et al.*, 2014 ▸; do Rosario *et al.*, 2014 ▸; Leib *et al.*, 2016 ▸). They consist of structures with varying refractive indices, typically spheres, but other shapes like rods are also possible (Wiersma, 2013 ▸). The size of the structures and the wavelength of the light need to be of roughly the same size.

Photonic properties like random lasing only emerge if the structure is clearly defined. In the case of photonic glasses, the particle arrangement has to be completely random. The requirements for thermal barrier coatings are more relaxed, but the structure still influences the performance and reflective properties (Sun *et al.*, 2000 ▸).

A profound knowledge of the three-dimensional inner structure is required in order to improve the fabrication and processing methods (García *et al.*, 2010 ▸; Kubrin *et al.*, 2013 ▸; do Rosario *et al.*, 2014 ▸; Dyachenko *et al.*, 2014 ▸; Leib *et al.*, 2016 ▸). This also applies for a better understanding of the the general relationship between structure and properties, especially the transition between ordered (photonic crystals) and disordered (photonic glasses) arrays of monodispersed spheres. The photonic glass investigated in this experiment consisted of zirconium oxide spheres of 2.05 µm diameter. Structures of this size can achieve a reflectivity of more than 80% over a broad range of the infrared spectrum (λ = 1–3 µm) (Dyachenko *et al.*, 2014 ▸). In order to cover a broader range of the infrared spectrum needed for applications as TBCs (λ = 1–5 µm), particles with diameters of about 2.60 µm can be used (Dyachenko *et al.*, 2014 ▸). The packing fraction is defined as the ratio of particle volume with respect to the total volume. For an accurate determination of the packing fraction, a resolution well below the diameter of the spherical components is required. Therefore, nanotomography is the ideal technique to study samples such as photonic glass with structure sizes of 2 µm. The high penetration depth of X-rays with energies above 15 keV allows measuring hard ceramic samples of well above 20 µm in diameter, allowing for good volume statistics in the sample. An X-ray microscopy setup has been realised at a photon energy of *E* = 17.4 keV using polymer X-ray optics fabricated by deep X-ray lithography. This type of optics can also be used for high energies of up to 30 keV (Marschall *et al.*, 2014 ▸), allowing investigations of strongly absorbing samples, for example nanoporous gold.

## 2. Experiment   

### 2.1. Sample preparation   

The sample consisted of a disordered arrangement of zirconium oxide spherical particles. The particles were synthesized *via* a modified sol-gel approach (Leib *et al.*, 2015 ▸). In a first step, nanometer-sized ZrO_2_ primary particles form in solution and then aggregate to microparticles. The size of the microparticles can be controlled by various synthetic parameters such as water content, zirconium precursor and stabilizer concentration (Leib *et al.*, 2015 ▸). The particles were separated by centrifugation. As-synthesized particles were pre-calcinated in two steps at *T*_1_ = 120°C and *T*_2_ = 450°C for 3 h each. The size of the resulting ZrO_2_ particles used for this sample was *d* = 2.05 ± 0.11 µm. Only after heating at *T* = 450°C did we find that the shrinkage and the mass loss in the zirconia particles due to decomposition of organic residue and densification stagnated. It was found that, for operation temperatures of above 1000°C, the particles had to be preheated in order to prevent shrinkage-inducing crack formation. For the assembly of a photonic glass film which does not shrink and subsequently cracks at operating temperatures of above 1000°C, the particles needed to be preheated (Leib *et al.*, 2015 ▸).

The preparation of the photonic glasses followed the process described by Dyachenko *et al.* (2014 ▸). Briefly, the pre-calcinated zirconia spheres were resuspended in ethylene glycol and the resulting suspension (300 mg ml^−1^ particles) was ultrasonicated for homogenization. A hydrophilic soda-lime glass was used as substrate and the area of the sample was defined by a silicon ring. The suspension was drop-cast on the substrate and the sample was then heated to *T* = 150°C to evaporate the solvent from the suspension. A variation of the sample thickness can be achieved by changing the concentration of the particles suspension cast into the area defined by the silicon ring. After evaporating the solvent, the layers were calcinated at *T* = 600°C for 2 h to remove residues of the ethylene glycol.

The drop-casting resulted in a thin layer of randomly arranged particles with a thickness *d* ≃ 35 µm. Since the contact area between the spheres and the resulting forces are very small, the overall stability of the coating is insufficient for handling and sample preparation. In order to stabilize the coating, a low-viscosity adhesive was used to infiltrate the sample and to fix the spheres in their respective positions: cyanoacrylate adhesive (Wiko SG15) was diluted with Leit-C thinner (Plano N651) and a drop of the mixture was applied to the sample, which was then dried at room temperature. While the topology of the sample could have been changed by capillary forces, scanning electron microscope (SEM) images of the sample surface showed no variation between original and treated regions of the sample.

**Figure 1:**
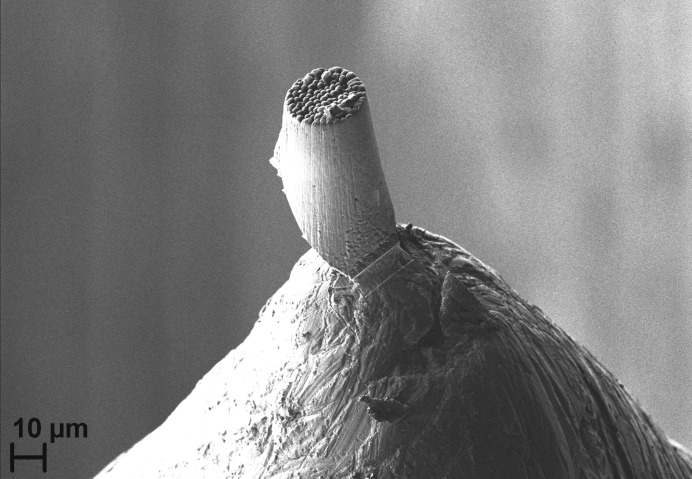
SEM image of the ZrO_2_ sample after preparation with the FIB and mounted on the sample holder.

Using a focused ion beam (Zeiss Auriga) working with gallium ions, a pillar of approximately 30 µm in diameter was prepared from the sample. The pillar was attached to a stainless steel sample holder using the cold soldering option of the FIB workstation (soldering material platinum). Fig. 1 ▸ shows an SEM image of the pillar on the sample holder.

### 2.2. Experimental setup   

The experiment was performed at the PETRA III P05 beamline (Haibel *et al.*, 2010*a* ▸,*b* ▸; Greving *et al.*, 2014 ▸) nanotomography endstation (Ogurreck *et al.*, 2013 ▸) in X-ray microscopy geometry. A double-crystal monochromator using silicon (111) reflections was used to select a monochromatic energy of *E* = 17.4 keV for the measurement.

The substructure of the experiment consists of a 6.8 m-long granite optical bench. All components in the experimental hutch are installed on four air-bearing sliders on this optical bench. These four sliders are primarily designated for condenser optics, sample, objective optics and X-ray detector. The condenser lens is installed on a PI H-824 hexapod on the first granite slider.

The sample stage with an air-bearing rotation axis is installed on the second granite slider. The rotation axis is specified for a position accuracy of below 50 nm. Measured axial and radial errors are 16.9 nm and 21.4 nm (both RMS), respectively. The measured wobble error is 0.22 µrad (RMS). The rotation axis is mounted on three pods for height adjustment and tip/tilt alignments. A linear translation stage mounted at the bottom of the sample stage allows for horizontal translations. The sample is positioned on the rotation axis *via* a six-axis kinematics mounted in the aperture of the rotation axis. The sample position is only 20 mm above the rotation plane, yielding very small displacement due to wobble errors. The sample can be aligned with respect to the axis of rotation using a six-axis kinematics installed in the aperture of the rotation stage.

The objective compound refractive lens (CRL) is installed on the third granite slider and aligned by an encoder-equipped PI miCos SpaceFab six-axis kinematics. Measured vibrations at the X-ray optics position are 7 nm (RMS). Apertures are installed in front of the CRL and mounted on slip-stick piezo motors.

The fourth slider is used for the detector system which consisted of a pco.4000 CCD camera in combination with an *M* = 20× infinity-corrected microscope objective and an *M* = 1× tube lens. A cerium-doped lutetium oxyorthosilicate crystal with a thickness *t* = 16 µm acted as scintillator. With this detector system an effective pixel size of 442 nm and a resolution of 1.6 µm was reached in the plane of the scintillator.

In this experiment, a rolled X-ray prism lens condenser (Vogt *et al.*, 2014 ▸) was used for creating an illumination spot of about 50 µm × 50 µm at a working distance of 1.2 m. A rotating paper disk was placed 1.05 m behind the condenser to act as diffuser (White *et al.*, 1995 ▸; Cloetens *et al.*, 1996 ▸; Morgan *et al.*, 2010 ▸) for homogenizing the illumination and reducing the degree of coherence. The sample was placed 0.15 m behind the diffuser disk.

**Figure 2:**
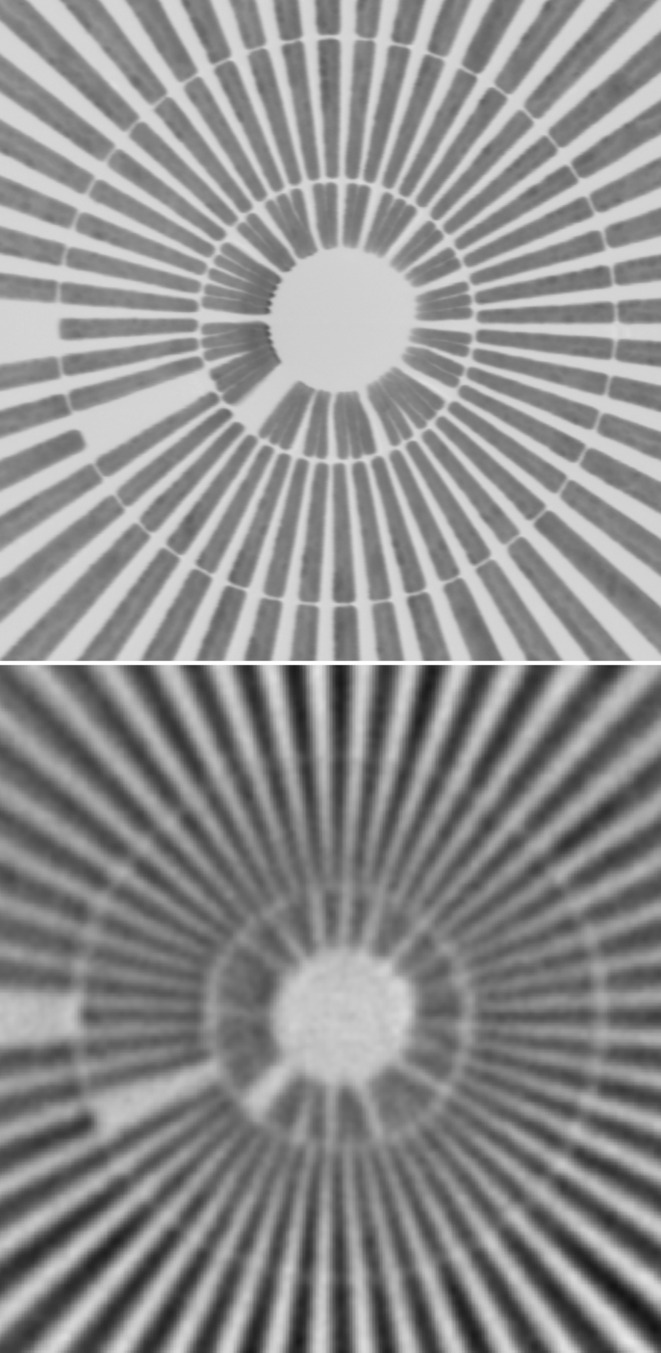
SEM (top) and XRM (bottom) images of the Siemens star test pattern. The line widths are 50–100 nm in the innermost circle and 100–200 nm in the second circle.

Gold apertures were installed behind the sample for defining the opening aperture of the objective lens. The objective optics used for this experiment were CRLs fabricated by deep X-ray lithography (Saile *et al.*, 2008 ▸; Reznikova *et al.*, 2008 ▸; Nazmov *et al.*, 2011 ▸) under ±45° on one substrate for horizontal and vertical focusing. The design used for this experiment was aperture-optimized lenses (Marschall *et al.*, 2014 ▸) with a focal distance *f* = 98.4 mm, consisting of *N* = 40 lens elements per direction. The geometric lens aperture is 98 µm and the theoretical resolution limit of the optics is 37 nm half-period. The resolution was determined using an Xradia test pattern X50-30-7Au. Fig. 2 ▸ shows the center of a Siemens star.

The detector was mounted 2.5 m behind the X-ray objective optics. A measurement of the effective detector pixel size yielded 17.2 nm in the sample plane, *i.e.* an X-ray magnification *M* = 25.7×. The resolution limit derived from these images was about 100 nm per half-period and in good agreement with data from modulation transfer function (MTF) calculations. The resolution determined by 10% visibility in the MTF was *r*_hor_ = 101 nm and 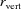 = 103 nm (half-period) at an effective pixel size of 17.2 nm.

**Figure 3:**
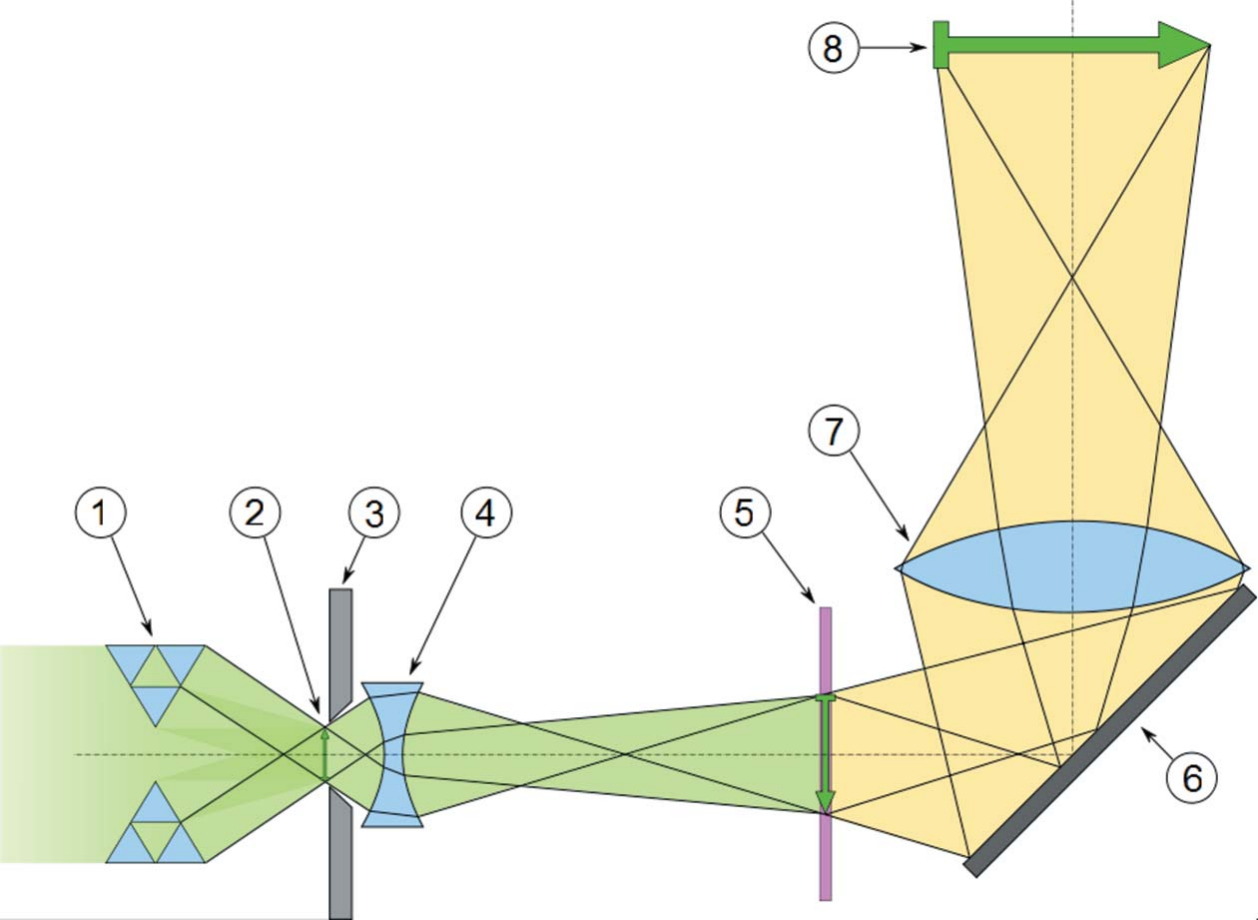
Schematic drawing of the optical layout used. The X-ray illumination of the sample is achieved by the rolled X-ray prism lens (1). The sample (2) is mounted in the focal plane of the CRL (4), which is protected from stray X-rays by the aperture (3). The scintillator (5) converts the X-rays to visible light and a tilted mirror (6) protects the microscope optics (7) from radiation damage. The sensor (8) captures the image (Marschall, 2014 ▸).

An overview of the optics layout is given in Fig. 3 ▸. The main advantage of using this kind of X-ray optics is the large flexibility. Key parameters such as field of view, working distance or resolution can be selected by fabricating tailored optics. Furthermore, these optics can be used over a wide range of X-ray energies, from well below 10 keV up to 30 keV (Marschall *et al.*, 2014 ▸), an energy which is not effectively achievable using Fresnel zone plates.

### 2.3. Experimental procedure   

**Figure 4:**
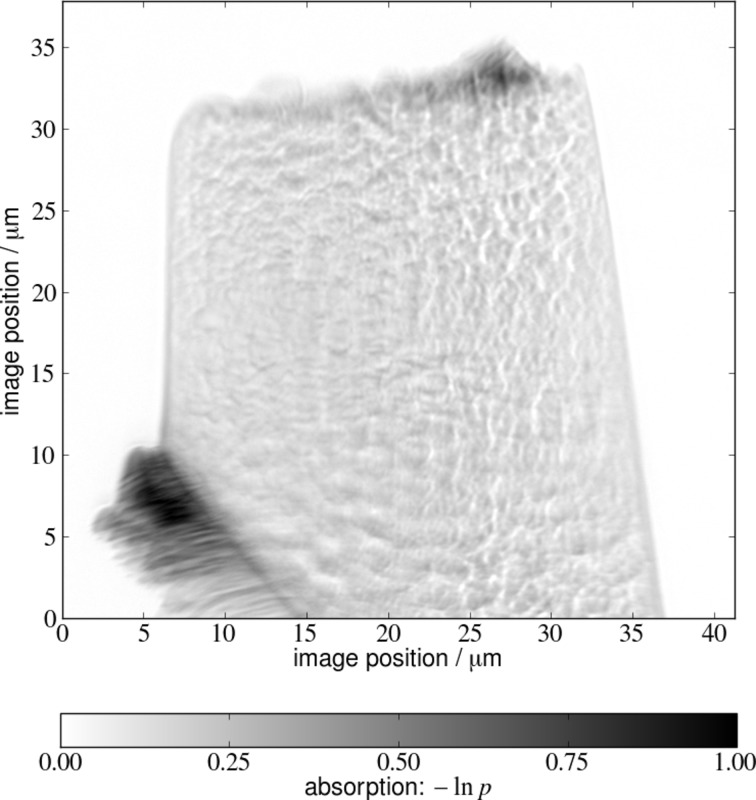
Normalized projection image. The higher absorption on the very top and in the bottom left corner originates from the sample preparation with the focused ion beam.

The photonic glass sample was measured with 450 angular steps over an angular range of 180°. Reference images were acquired every second angular step. All images were corrected for camera dark current and the storage ring electron beam intensity, which varies about 1% even in top-up operation. To account for beam position variations, the sample projections and all reference images were correlated in an image region which was never shaded by the sample. The best fitting image was selected for background correction. The images were registered for position drifts of sample and objective optics by tracking sample features and the sample outline. Images were further processed with a binning of 4, *i.e.* an effective pixel size of 68.8 nm in the sample plane. An exemplary projection image is given in Fig. 4 ▸, showing the sample outline and the contrast of the sample. The contrast level with a maximum absorption value of roughly 70% is below what would be ideally used in a tomography, but the selected contrast level is a compromise between high contrast in the sample and a high transmission of the experiment.

**Figure 5:**
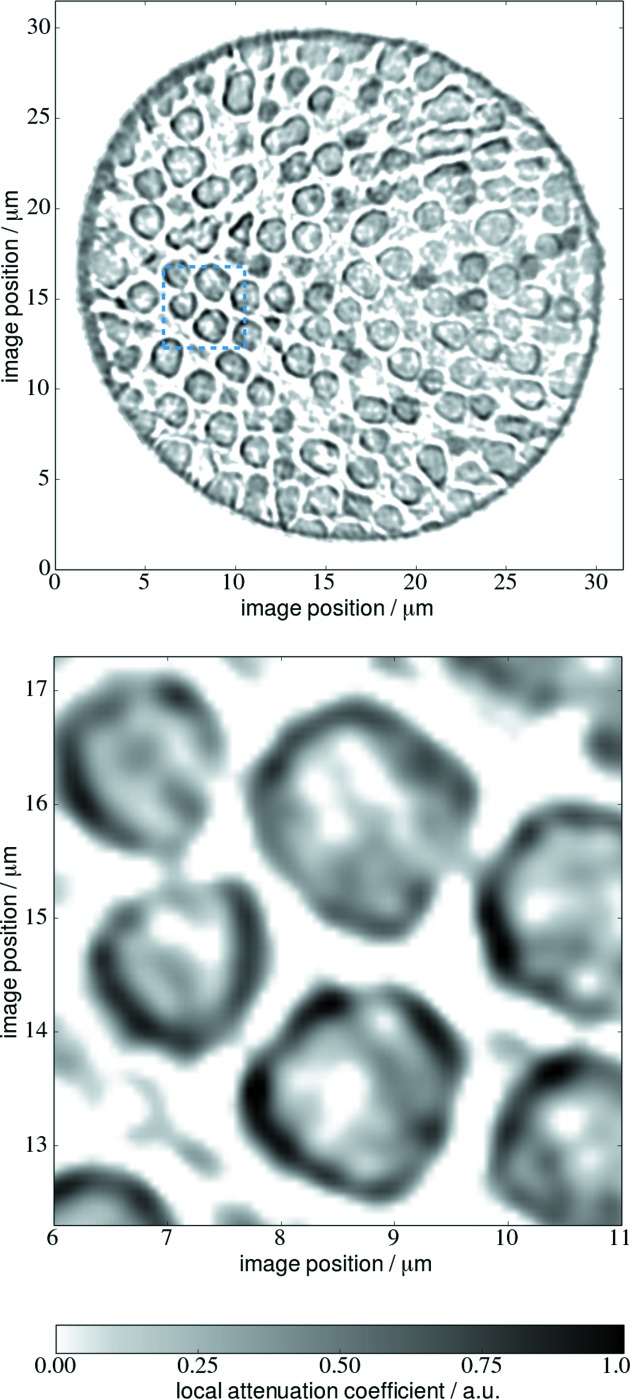
Reconstructed slice of the zirconia photonic glass sample (top). A magnified view of the blue dotted square is shown at the bottom. There are still phase artifacts visible in the reconstruction, for example edge enhancements but the overall sample structure is clearly visible.

**Figure 6:**
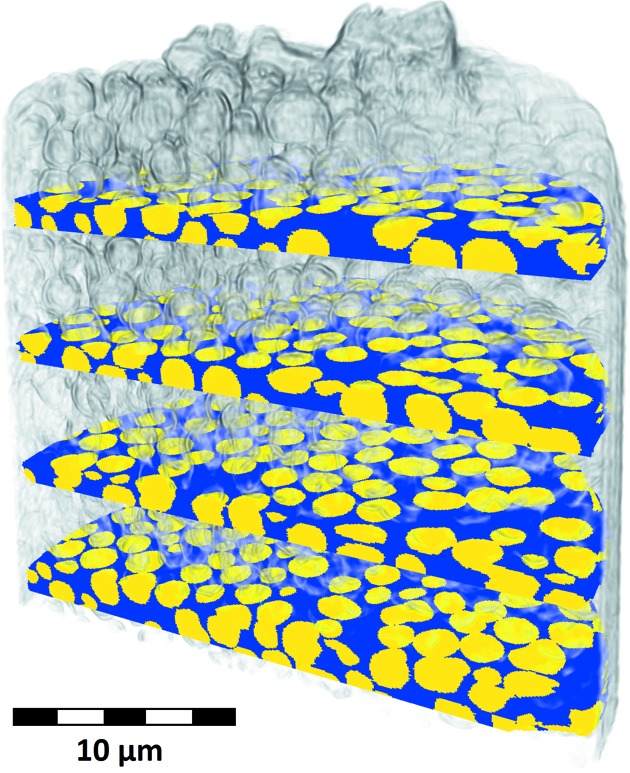
Visualization of the segmented photonic glass sample. The zirconia particles are marked in yellow whereas the empty matrix is shown in dark blue. A transparent overlay of the sample outline shows the positions of the analyzed areas with respect to the sample.

A filtered backprojection algorithm was used for the reconstruction. A reconstructed slice is shown in Fig. 5 ▸. The sample preparation using focused ion beam (FIB) milling is responsible for material redeposition close to the sample outline. This shows as a dark and dense outer layer in the sample. Segmentation of the data set was performed manually using *Avizo Fire* (version 8.0) at four heights in the sample at a mean spacing of 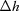 = 6.88 µm, corresponding to 100 slices offset. At each of these positions, seven slices with a slice spacing of 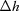 = 206.4 nm were analyzed. A visualization of the segmented data is shown in Fig. 6 ▸.

## 3. Results and discussion   

The nanotomography measurement was performed to achieve a three-dimensional quantification of the mean packing fraction.

**Figure 7:**
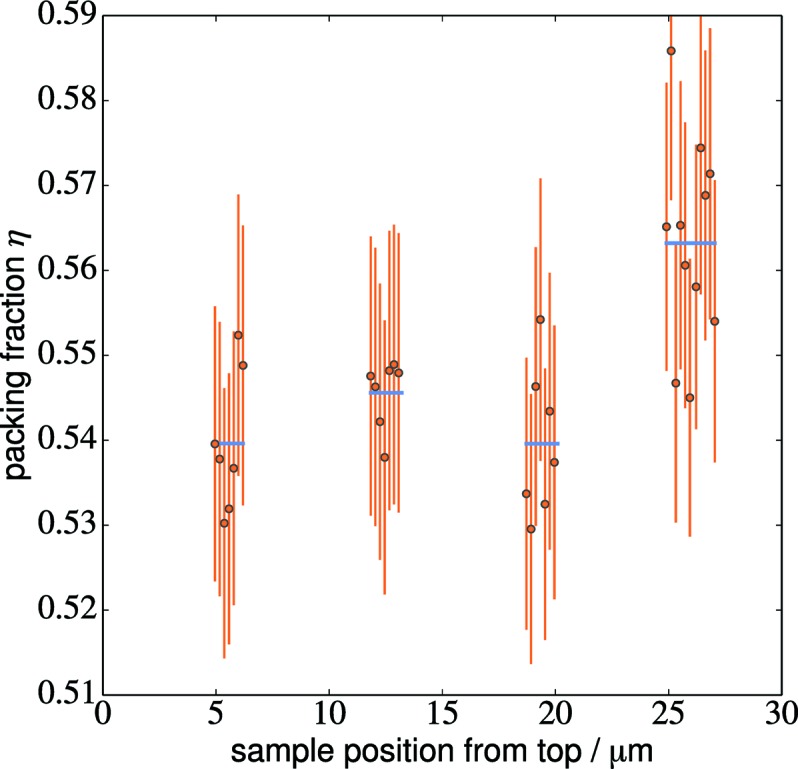
Plot of the packing fraction η for the photonic glass sample. Individual slices are marked with orange dots and error bars, local ensemble averages are given by the blue bars. The packing fraction is very constant throughout the sample height with an increase only towards the bottom.

**Figure 8:**
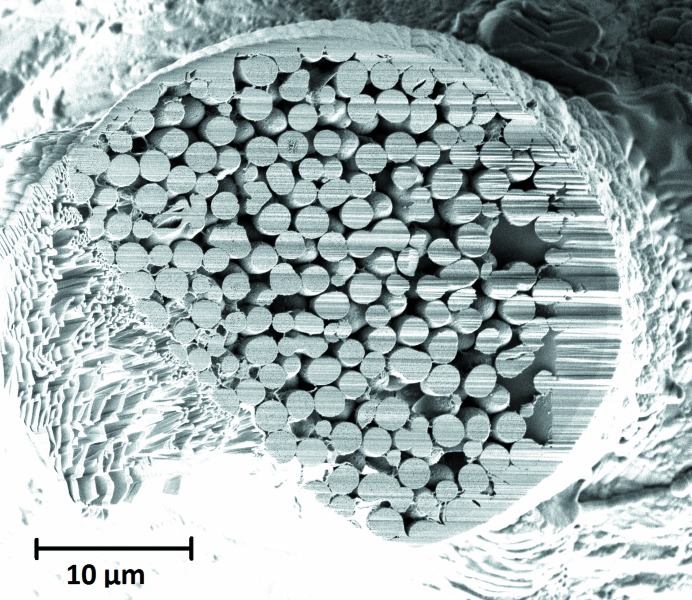
SEM image of the FIB slicing surface used for determination of the packing fraction. FIB curtaining effects can be seen in the right part of the sample and are caused by the large sample size and ion beam defocusing.

Because of material redeposition during the FIB milling, the sample was covered by a dense material layer and the outermost part of the sample was ignored for determining the packing fraction. The measured mean packing fraction in the sample is η = 0.549 ± 0.003. Fig. 7 ▸ shows how the packing fraction varies throughout the sample volume. The errors shown in Fig. 7 ▸ are estimated errors from the manual segmentation; the overall error in the packing fraction is the standard error of the estimate. The data show a significant increase in the packing fraction towards the lower end of the sample. The average packing fraction of the upper 20 µm of the sample is η = 0.542 ± 0.006 whereas the bottom region yields η = 0.563 ± 0.008. The sample was sliced with a focused ion beam after the synchrotron tomography experiment in the lower part of the sample. An SEM image was used to confirm the local packing fraction. A value η = 0.562 ± 0.008 was measured which is in very good agreement with the nano­tomography data for the lower region of the sample. Fig. 8 ▸ shows the SEM image used for calculating the packing fraction.

There is no apparent global density gradient throughout the sample, but a local change in the density: the packing fraction is highest at the bottom of the sample. Because of the rigid glass substrate, the first layer of particles is slightly more packed in their respective height and more disorder only emerges with the next layers of particles. The overall average packing fraction η = 0.549 ± 0.006 is significantly smaller than a random close packing of η ≃ 0.64 (Finney, 1970 ▸), which is the highest possible packing fraction for spheres without long-range order. Sapienza *et al.* (2007 ▸) estimated the packing fractions in similarly arranged samples to η = 0.55, which is in good agreement with the numbers found for these samples. In the literature, values obtained for random packing densities depend on the forces that act during packing (Jaeger & Nagel, 1992 ▸; Torquato *et al.*, 2000 ▸). These forces are dominated by gravity, friction and elastic repulsion but, when particles in a suspension are sedimenting, gravity is weakened by the buoyancy of the particles. Onoda & Liniger (1990 ▸) experimentally found a connection between the packing density and acting forces, *i.e.* gravity, when sedimenting spheres from a suspension. Under ambient conditions they found a packing fraction η ≃ 0.595. In the limit of zero force, *i.e.* an equal mass density of particles and solvent, an experimental limit of η = 0.555 ± 0.005 was obtained. These numbers are in good agreement with the packing fraction obtained for the photonic glass sample prepared by drop-cast suspensions.

Comparing the packing fraction of these random arrangements with ordered structures is of interest because the fabrication process of photonic crystals and photonic glasses is very similar. It is possible to create samples that show photonic crystalline as well as photonic glassy behaviors alternatingly over the length of the sample (Emoto & Fukuda, 2012 ▸). Small variations in the ordering and packing fraction induce a variation of the local structure resulting in a change of the photonic properties from crystalline to glassy. Therefore a correlation of the structure to the processing conditions is essential for fabricating photonics with tailored properties. This is especially true when working with monodisperse particles as these will very easily form ordered structures (García *et al.*, 2007 ▸, 2010 ▸).

In order to achieve stable structures during the sedimentation process, the particles need to be jammed, *i.e.* unable to collapse. Although it is possible to create ordered jammed arrangements with packing densities η < 0.1 (Torquato & Stillinger, 2007 ▸), these arrangements will not appear in self-organizing assembly. A face-centered cubic (f.c.c.) structure with η = 0.74 is the upper limit for the spherical packing fraction. Starting with this lattice structure and randomly removing individual particles, the f.c.c. structure will remain stable until the packing fraction falls below η ≃ 0.52. Then, the ordered structure will collapse and a random arrangement will occur. Such a structure could explain why ordered and dis­ordered regions occur from very similar starting conditions. Assuming that an ordered first layer is formed, the hexagonal structure will collapse and a random packing will take place if the further sedimentation occurs too fast and leaves too many voids. Otherwise, if a large number of voids are incorporated in an ordered hexagonal packing, the photonic response of the system will still correspond to a photonic glass (García *et al.*, 2007 ▸).

## 4. Conclusion   

A nanotomography experiment was performed at the beamline P05 for determining the packing fraction in a photonic glass. The photon energy of 17.4 keV allowed the investigation of a zirconia sample of 30 µm diameter at a resolution of 103 nm half-period. The reconstruction was performed using a filtered backprojection algorithm. Unlike surface-sensitive techniques like SEM, X-ray nanotomography gives detailed information about the bulk structure of the sample. The acquired three-dimensional data set of the sample contains many additional information like density distribution and packing fraction. The investigation of structural gradients, *e.g.* a gradient in the packing fraction, within the sample is also only possible with volume information.

Analyzing the photonic glass at different heights, a very homogeneous global packing fraction was determined throughout the sample depth. The packing fraction has been confirmed by using a FIB and SEM imaging. The obtained value 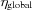 = 0.549 ± 0.003 is consistent with the expected packing fraction for a force-free sedimentation of spherical particles. It is, however, significantly smaller than a random close packing with η = 0.64, which would be the ideal case for disordered structures. Using the corrected packing fraction, simulations of the photonic properties of this sample can be improved. If the photonic properties require a higher packing fraction, the experimental procedure for the fabrication of these photonic glasses can be adapted, for example by using higher forces during sedimentation.
